# Risk and protective factors associated with being bullied on school property compared with cyberbullied

**DOI:** 10.1186/s12889-016-2833-3

**Published:** 2016-02-12

**Authors:** Ray M. Merrill, Carl L. Hanson

**Affiliations:** Department of Health Science, College of Life Sciences, Brigham Young University, 2063 Life Sciences Building, Provo, Utah 84602 USA

## Abstract

**Background:**

We identified bullying victimization (bullied on school property versus cyberbullied) by selected demographic, personal characteristic, and behavior variables.

**Methods:**

A cross-sectional analysis was conducted on adolescents (*n* = 13,583) completing the 2013 Youth Risk Behavior Survey (YRBS) in grades 9 through 12.

**Results:**

Being bullied on school property in the past 12 months was significantly more common in females than males, in earlier school grades, and in Whites and other racial groups compared with Blacks and Hispanics. Being bullied on school property generally decreased with later school grades, but cyberbullying in the past 12 months remained constant. Being bullied on school property or cyberbullied was significantly positively associated with mental health problems, substance use, being overweight, playing video games for 3 or more hours per day, and having asthma. The association was greatest with having mental health problems. Cyberbullying was generally more strongly associated with these conditions and behaviors. Protective behaviors against bullying victimization included eating breakfast every day, being physically active, and playing on sports teams. Those experiencing victimization on school property and cyberbullying were significantly more likely to experience mental health problems compared with just one of these types of bullying or neither.

**Conclusions:**

Cyberbullying victimization is generally more strongly associated with mental health problems, substance use, being overweight, playing video games for 3 or more hours per day, and having asthma than bullying victimization on school property. However, because bullying on school property is more common in grades 9–11, this form of bullying has a greater burden on these conditions and behaviors in these school grades.

## Background

Recent adolescent suicides and school shootings have received heightened publicity and raised awareness regarding the growing public health problem of bullying. Bullying is prevalent and often has detrimental effects on individuals, families, and communities [1]. While definitions of bullying vary, most researchers agree it involves targeted intimidation or humiliation [2], and includes (1) aggressive and intentionally abusive behavior towards another that (2) persists over time, and (3) consists of an imbalance of power between the perpetrator and victim [3–7]. Although bullying occurs among young children and adults, the majority of research focuses on adolescents in schools [2]. Studies report that approximately 10–30 % of adolescents are involved as bullies, victims, or both bullies and victims [4], which is consistent throughout the United States [8], Europe [9, 10], Latin America [11–13], and Australia [14].

Being a victim of bullying can be classified as direct or indirect [15, 16]. Direct forms of bullying consist of physical attack and/or verbal harassment, whereas indirect forms of bullying involve social exclusion, spreading rumors and other related, passive-aggressive actions [7, 15, 17–19]. In other words, direct forms of bullying involve intimidating, humiliating, or belittling, whereas indirect forms seek to destroy an individual’s social reputation and/or status while concealing the identity of the perpetrator [2]. Numerous studies confirm that direct confrontation does not progress to indirect forms of bullying with age [20]. Cyberbullying is viewed as an extension of traditional bullying and includes the use of electronic or digital media by individuals or groups to communicate hostile or aggressive text messages or emails, embarrassing pictures and rumors posted on social networking sites, and distorted profiles intended to inflict harm or discomfort on others [21]. It differs from traditional forms of bullying in that the aggressor often remains anonymous [22].

Bullying among adolescents can have adverse effects on the victims in terms of distress, as well as subsequent deviant behaviors and possible persistent psychiatric problems [9, 14, 23–25]. For example, one study of sixth, ninth, and twelfth grade students in Minnesota found that 6.1 % reported frequent (weekly in the past 30 days) perpetration only, 9.6 % frequent victimization only, and 3.1 % both, with suicidal thinking for these three groups being 22 %, 29 %, and 38 %, respectively [26]. Research has found that bullying victims experience greater levels of fear and worry, disobedience, lying, and irritability [27], as well as higher levels of insecurity, anxiety, and depression [28–31]. Bullying victims tend to internalize behaviors such as depression, anxiety, and social withdrawal while externalizing behaviors such as disruptiveness, dishonesty, and aggression [32, 33].

In an effort to better address bullying victimization, researchers have sought to find the antecedents to victimization by identifying the predictors. These predictors are typically referred to as risk and protective factors and are often assessed in a school setting. Risk factors are behaviors or characteristics that predict a future problem while protective factors are those indicators that reduce or prevent a future problem [34]. Risk and protective factors of bullying victimization may differ according to whether the form of bullying is on school property or cyberbullying through e-mail, chat rooms, instant messaging, websites, or texting.

The purpose of this study was to compare victimization levels of bullying on school property and cyberbullying among students in grades 9 through 12 across selected demographic, personal characteristic, and behavior variables. The association between experiencing mental health problems and the frequency of those problems with being bullied or cyberbullied was also explored. Finally, we will assess the frequency of mental health problems according to combinations of being bullied and/or cyberbullied.

## Methods

### Sampling

The Youth Risk Behavior Surveillance System (YRBSS) monitors health and protective behaviors among youth in the United States, such as mental health, substance use, sexual behavior associated with unintended pregnancy and sexually transmitted infections, body weight, and physical activity/inactivity. The prevalence of obesity and asthma are also monitored. The YRBSS includes the national school-based Youth Risk Behavior Survey (YRBS), administered by the Centers for Disease Control and Prevention (CDC), as well as urban school district school-based YRBSS, administered by state and local education and health agencies. Public and private schools from all 50 states and the District of Columbia are represented in the YRBS.

The sample design involved a three-stage cluster sampling process in order to obtain a nationally representative sample of students attending public and private schools in grades 9 through 12. In the first stage the sampling frame consisted of 1,276 primary sampling units (PSUs), which involved counties, subareas of large counties, and groups of smaller, adjacent counties. From the PSUs, a sample of 54 was taken, based on probability sampling proportional to the overall school enrollment size for the PSU. The second stage of sampling consisted of 193 schools with any grade 9 through 12, based on probability proportional to the size of the school enrollment. The third stage of sampling involved random sampling within each grade, of which one or two classrooms were chosen from a required subject or period. Black and Hispanic students were oversampled. Additional details of the three sampling scheme and the strategies for oversampling Blacks and Hispanics can be found elsewhere [35].

### Data collection and questionnaire

Participation in the survey was anonymous and voluntary. Prior to participating in the survey, parental permission was obtained. Completion of the questionnaire was carried out in a single class period and followed the CDC’s Institutional Review Board protocol for the YRBS. The survey takes approximately 40 min to complete. Students whose parents did not provide consent participated in an alternate reading activity, while their classmates completed the survey. The questionnaire consisted of 86 questions and is available online [36]. Studies have assessed and improved the questionnaires validity and reliability since the surveys inception in 1991 [37]. The anonymous survey was deemed exempt from institutional review board review at Brigham Young University.

### Data and response rates

The 2013 YRBS consists of 13,633 questionnaires from 148 public and private schools, 50 of which failed quality control and were excluded, leaving 13,583 for assessment. Response rates were 77 % for the schools, 88 % for the students, and 66 % overall [38]. Additional details of the data processing procedures and response rates are available elsewhere [38].

### Variables

Two outcome variables involved questions about bullying. Prior to asking these two questions on the YRBS, the following statement was made on the instrument: “Bullying is when 1 or more students tease, threaten, spread rumors about, hit, shove, or hurt another student over and over again. It is not bullying when 2 students of about the same strength or power argue or fight or tease each other in a friendly way.” The first bullying question asked: “During the past 12 months, have you ever been bullied on school property?” The second bullying question asked: “During the past 12 months, have you ever been electronically bullied? (Count being bullied through e-mail, chat rooms, instant messaging, websites, or texting.)”

Independent variables consisted of demographic variables include age (12 years or younger, 13 years, 14, years, 15 years, 16 years, 17 years, and 18 years or older), sex, race/ethnicity (White, Black or African American, Hispanic/Latino, All other races), and grade (9, 10, 11, 12), and selected mental health, substance use, sexual behavior weight, and other variables shown in Table 3. Race/ethnicity was derived from two questions: (1) “Are you Hispanic or Latino?” (response options “yes” or “no”) and (2) “What is your race?” (response options “American Indian or Alaska Native,” “Asian,” “Black or African American,” “Native Hawaiian or other Pacific Islander,” or “White).

### Statistical assessment

Cross-tabulations were evaluated using the Rao-Scott chi-square test. Slope coefficients from regression analysis were assessed using the t test. Statistical significance was based on two-tailed hypothesis tests at the 0.05 level. The YRBS employs a complex sampling scheme, which requires that we account for the sampling design (stratification, clustering, and unequal selection probabilities) in order to obtain valid estimates and tests of hypotheses. Analyses were conducted using the Statistical Analysis System, version 9.4 (SAS Institute Inc., Cary, NC, USA, 2012) procedures SURVEYFREQ and SURVEYREG, which are designed to analyze the complex sample survey data correctly; tratum, primary sampling unit, and weight variables were included in the data set and accounted for in the SAS procedures. The weight variable was based on student sex, race/ethnicity, and grade. It was applied to each record to adjust for oversampling of Black and Hispanic students and for school and student nonresponse. The weighted estimates represent all public and private school students in the United States in grades 9 through 12.

## Results

**Fig. 1:**
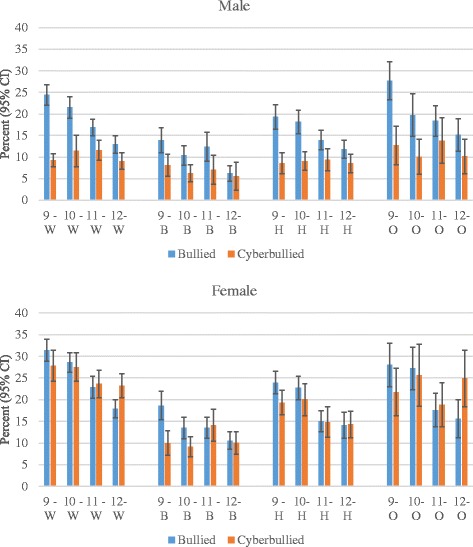
Bullied on school property or cyberbullied by sex, school grade, and race/ethnicity (White [W], Black [B], Hispanic [H], Other [O])

Being bullied on school property in the past 12 months was significantly more common in females than males, in earlier school grades, and in Whites and other racial groups compared with Blacks and Hispanics (Table 1). Being bullied on school property compared with cyberbullied in the past 12 months was more common in males, grades 9–11, and across the racial/ethnic groups, more so among Blacks and Hispanics. Among males and females, being bullied on school property generally decreased with later school grades, but cyberbullying remained somewhat constant across school grades within each racial/ethnic group (Fig. 1). Frequency of being a victim of cyberbullying was more similar to being bullied on school property for females, and even tended to surpass it in grades 11 and 12 for Whites and other racial groups.

**Table 1:** Bullying Victimization According to Selected Demographic Variables

			During the past 12 months, have you ever been bullied on school property?			During the past 12 months, have you ever been electronically bullied? (Count being bullied through email, chat rooms, instant messaging, websites, or texting.)			Bullied to Cyber-bullied Ratio
	Weighted Number	Weighted %	%	Estimate	95 % CI	%	Estimate	95 % CI	Ratio
Sex									
Male	88704	51.3	17.61	1.00	Referent	9.69	1.00	Referent	1.82
Female	84154	48.7	22.27	1.26	1.20–1.33	21.57	2.26	2.04–2.43	1.03
Grade									
9^th^	46795	27.1	24.56	1.75	1.61–1.89	15.79	1.11	1.00–1.23	1.56
10^th^	43948	25.5	22.02	1.57	1.45–1.70	16.33	1.15	1.02–1.28	1.35
11^th^	41331	24.0	17.60	1.25	1.14–1.37	15.42	1.08	0.96–1.22	1.14
12^th^	40394	23.4	14.05	1.00	Referent	14.23	1.00		0.99
Race/Ethnicity									
White	107293	62.7	22.07	1.00	Referent	17.79	1.00	Referent	1.24
Black	23996	14.0	12.69	0.58	0.53–0.63	8.81	0.50	0.44–0.56	1.44
Hispanic/Latino	24117	14.1	17.96	0.81	0.75–0.88	13.19	0.74	0.66–0.84	1.36
All other races	15795	9.2	21.68	0.98	0.89–1.08	17.25	0.97	0.85–1.10	1.26

Data source: Youth Risk Behavior Survey (YRBS), 2013

Being a victim of bullying within each school grade was greatest in the most common age in that grade, for both bullied on school property and cyberbullying (Table 2). Yet the age immediately younger than the most common age within each grade had a much higher level of bullying victimization than the age immediately older than the most common age. Within each grade, there is no consistent pattern across age in the ratio of being bullied compared with cyberbullied, with the exception of the ratio being greatest in the age group just prior to the most common age.

**Table 2:** Bullying Victimization According to School Grade and Age

Grade	Age	Weighted No.	Weighted %	Bullied %	95 % CI	Cyber-bullied %	95 % CI	Bullied to Cyber-bullied Ratio
9th	12 year or younger	43	0.09	0.06	0.01–0.10	0.06	0.00–0.14	1.00
	13 years	151	0.32	0.08	0.02–0.15	0.06	0.00–0.14	1.33
	14 years	17895	38.27	10.34	9.48–11.20	6.12	5.31–6.93	1.69
	15 years	24641	52.69	12.58	11.55–13.61	8.37	7.34–9.39	1.50
	16 years	3598	7.69	1.26	0.97–1.55	0.99	0.67–1.31	1.27
	17 years	326	0.70	0.18	0.09–0.27	0.11	0.01–0.21	1.64
	18 years or older	112	0.24	0.05	0.00–0.11	0.07	0.00–0.16	0.71
10th	13 year or younger	26	0.06	0.03	0.00–0.06	0.03	0.00–0.07	1.00
	14 years	141	0.32	0.13	0.00–0.26	0.10	0.00–0.23	1.30
	15 years	17232	39.24	8.69	7.95–9.43	6.04	5.09–6.98	1.44
	16 years	23548	53.62	12.10	11.02–13.18	9.39	8.17–10.62	1.29
	17 years	2710	6.17	1.02	0.74–1.29	0.76	0.48–1.04	1.34
	18 years or older	257	0.58	0.06	0.01–0.11	0.02	0.00–0.05	3.00
11th	14 year or younger	39	0.09	0.08	0.01–0.14	0.04	0.00–0.10	2.00
	15 years	181	0.44	0.07	0.01–0.13	0.08	0.01–0.14	0.88
	16 years	17508	42.38	7.56	6.80–8.33	6.03	5.02–7.05	1.25
	17 years	21261	51.46	9.20	8.32–10.08	8.60	7.71–9.50	1.07
	18 years or older	2327	5.63	0.69	0.51–0.87	0.67	0.42–0.91	1.03
12th	15 year or younger	87	0.22	0.06	0.01–0.11	0.13	0.04–0.21	0.46
	16 years	272	0.67	0.08	0.01–0.14	0.11	0.02–0.20	0.73
	17 years	17907	44.37	6.38	5.61–7.15	6.13	5.24–7.02	1.04
	18 years or older	22095	54.74	7.51	6.82–8.20	7.87	6.98–8.75	0.95

Data source: Youth Risk Behavior Survey (YRBS), 2013

Being bullied on school property in the past 12 months was significantly greater across the mental health, substance use, weight, and asthma variables, more so for the mental health variables (Table 3). Among the sexual behavior variables, only having had sex before 13 years of age was significantly, positively associated with bullying victimization. Playing video games 3+ hours per day was also positively associated with bullying victimization. Behaviors associated with significantly lower bullying victimization were eating breakfast on all of the past 7 days, active 60 min on 5 or more of the past 7 days, and playing on 1 or more sports teams in the past 12 months. Cyberbullying in the past 12 months was also significantly greater across the mental health, substance use, sexual behavior, weight, and asthma variables, again more so for the mental health variables. Playing video games 3+ hours per day was also positively associated with cyberbullying victimization. Behaviors associated with significantly lower cyberbullying victimization were eating breakfast on all of the past 7 days and active 60 min on 5 or more of the past 7 days. Contrary to being bullied on school property, cyberbullying victimization was not associated with playing on 1 or more sports teams in the past 12 months.

**Table 3:** Bullying Victimization According Mental Health, Substance Use, Sexual Behavior, Weight, Selected Behaviors, and Asthma

	%	Bullied among Cases %	Bullied among non-Cases	Cases to non-cases Bullied Ratio	95 % CI	Cyber-bullied among Cases %	Cyber-bullied among non-Cases	Cases to non-cases Cyber-bullied Ratio	95 % CI	Bullied to Cyber-bullied Ratio
Mental Health										
Sad 2 wks past 12 mos	28.06	33.68	14.47	2.33	2.20–2.46	30.16	9.52	3.17	2.94–3.42	1.21
Considered suicide 12 mos	15.40	39.38	16.25	2.42	2.29–2.56	35.17	11.60	3.03	2.81–3.27	1.19
Made suicide plan 12 mos	12.31	40.53	16.93	2.39	2.27–2.52	35.16	12.51	2.81	2.60–3.04	1.21
Attempted suicide 1+ times 12 mos	7.26	43.84	18.43	2.38	2.23–2.54	41.66	13.64	3.05	2.83–3.29	1.13
Suicide attempt w/injury 12 mos	2.30	48.87	19.63	2.49	2.26–2.74	47.91	15.09	3.18	2.78–3.63	1.09
Substance Use										
Smoked 1+ past 30 days	18.00	24.42	18.72	1.30	1.22–1.40	23.86	13.50	1.77	1.60–1.95	1.15
Smoked cigarette before 13	10.11	26.21	19.02	1.38	1.28–1.49	21.90	14.59	1.50	1.34–1.68	1.24
Had 1+ drinks past 30 days	38.84	21.31	18.60	1.15	1.07–1.22	20.38	12.12	1.68	1.52–1.86	1.23
Had first drink before 13	20.06	24.76	18.79	1.32	1.24–1.40	19.41	14.78	1.31	1.22–1.42	1.27
Used marijuana 1+ times past 30 days	22.45	21.07	19.46	1.08	1.02–1.15	20.68	13.86	1.49	1.37–1.62	1.17
Tried marijuana before 13	7.96	24.18	19.48	1.24	1.14–1.35	20.28	15.03	1.35	1.21–1.50	1.24
Took steroids 1+ times in life	3.33	33.73	19.42	1.74	1.56–1.93	32.93	14.95	2.20	1.94–2.50	1.11
Sexual Behavior										
Had sex ever	46.80	19.91	19.79	1.01	0.94–1.07	19.41	12.04	1.61	1.48–1.75	1.26
Had sex before 13	5.91	23.13	19.65	1.18	1.07–1.30	17.97	15.37	1.17	1.01–1.35	1.28
Had sex with 4+ people in life	14.72	19.61	19.90	0.99	0.92–1.05	22.21	14.33	1.55	1.40–1.71	1.08
Had sex with 1+ people 3 mos	34.09	19.62	19.99	0.98	0.92–1.05	20.32	13.04	1.56	1.44–1.68	1.19
Weight										
Slightly/very overweight	29.18	24.11	18.13	1.33	1.26–1.40	18.48	14.30	1.29	1.19–1.40	1.29
Trying to lose weight	45.99	22.89	17.33	1.32	1.26–1.38	19.37	12.22	1.59	1.47–1.71	1.27
Fasted to lose weight past 30 days	11.86	34.54	17.88	1.93	1.80–2.07	32.78	13.10	2.50	2.32–2.70	1.14
Took pills to lose weight past 30 days	5.01	35.54	19.05	1.87	1.72–2.02	34.54	14.55	2.37	2.14–2.63	1.11
Vomited to lose weight past 30 days	4.19	37.23	19.07	1.95	1.80–2.12	40.52	14.45	2.81	2.56–3.07	1.02
Selected Behaviors										
Ate breakfast on all of the past 7 days	37.93	18.51	21.20	0.87	0.81–0.94	12.77	17.58	0.73	0.67–0.79	1.31
Active 60 min on 5+ past 7 days	48.47	18.80	20.99	0.90	0.84–0.96	14.12	16.97	0.83	0.78–0.89	1.28
Watched 3+ hours of TV average day	32.57	20.36	19.65	1.04	0.98–1.10	14.76	15.95	0.93	0.85–1.01	1.30
Played video games 3+ hours/day	31.99	22.99	18.40	1.25	1.18–1.32	18.46	13.93	1.32	1.23–1.43	1.28
Played on 1+ sports teams 12 mos	57.09	19.31	20.75	0.93	0.89–0.98	15.49	15.69	0.99	0.91–1.07	1.28
Asthma										
Told by doctor/nurse they had asthma	21.97	23.87	18.80	1.27	1.21–1.34	19.80	14.34	1.38	1.26–1.51	1.25

Data source: Youth Risk Behavior Survey (YRBS), 2013

**Fig. 2:**
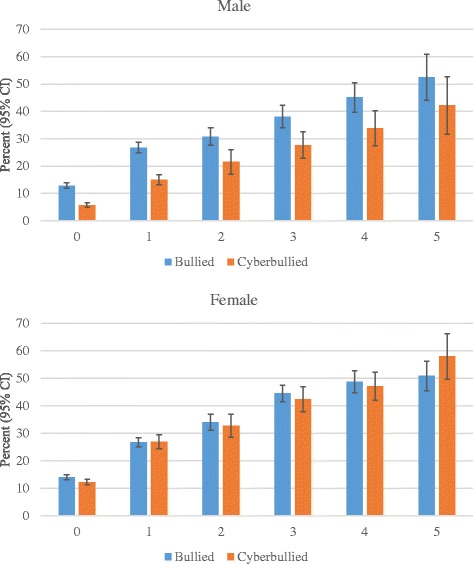
Bullied or cyberbullied by frequency of mental health issues and sex

The frequency of mental health issues (0 through 5) was positively associated with both being bullied on school property in the past 12 months and being cyberbullied in the past 12 months for both males and females (Fig. 2). In a regression model, frequency of mental health issues was regressed on a new variable (both bullied and cyberbullied, bullied only, cyberbullied only, neither bullied or cyberbullied), adjusting for age, sex, and race/ethnicity. This model estimated the average frequency of mental health issues as 0.21 (SE = 0.04) for neither bullied or cyberbullied, 0.87 (SE = 0.08, *p* < 0.0001) for cyberbullied only, 0.72 (SE = 0.07, *p* < 0.0001) for bullied only, and 1.38 (SE = 0.09, *p* < 0.0001) for both bullied and cyberbullied. The latter three *p* values indicate that the averages are significantly greater than the average frequency of mental health issues for neither bullied or cyberbullied.

## Discussion

This study identified bullying victimization by selected demographic, personal characteristic, and behavior variables. Differences in types of bullying victimization (bullied on school property and cyberbullied) were also assessed across the variables. Mental health problems associated with bullying and/or cyberbullying victimization were also explored.

Greater risk of being bullied among females than males is consistent with previous studies [39–42]. However, the risk of being cyberbullied compared with bullied on school property was noticeably more pronounced in females, which is also consistent with other research [43, 44]. A large, nationally representative survey conducted in the United States in 2013 found that the higher risk of cyberbullying among females persisted for many types of cyberbullying (i.e., hurtful information on the internet, private information purposely shared on the internet, subject of harassing instant messages, subject of harassing text messages, and subject of harassing e-mails) [44]. Although being bullied on school property generally decreased with later school grades for males and females, cyberbullying remained more constant within each racial/ethnic group. Higher levels of cyberbullying victimization among females may be because they more likely choose to bully other females on an emotional level through indirect means where they can remain anonymous in order to not jeopardize closeness and intimacy in their peer group, which is generally more important for girls than boys [22].

Blacks and Hispanics were the least likely to be bullied, as consistent with results from two large, nationally representative samples in the United States [41, 45]. The latter study found that Blacks were less likely than Whites to be victims of verbal or relational bullying [41]. In another national report compiled in 2013, the frequency of students aged 12–18 who indicated being bullied during the school year was 24 % for Whites, 20 % for Blacks, 19 % for Hispanics, and 9 % for Asians [43]. It may be that Blacks, Hispanics, and Asians are less willing than Whites to be victimized or appear less vulnerable. Further, being bullied on school property was more common than cyberbullying for each of the racial/ethnic groups, although more so for Blacks and Hispanics. This is consistent with lower levels of Internet and computer use among Blacks and Hispanics than Whites or Asians [46].

Previous research has also identified a decrease in traditional bullying victimization with increasing age and school grade [47–51]. Studies have shown that bullying victimization (primarily physical forms) peak at the end of middle school and the beginning of ninth grade, but decline thereafter [51–54]. Two possible reasons for the decrease are that younger children have more children around who might bully them, and the social skills of possible victims of bullies improves as they get older [55]. In contrast, other research has shown that verbal and cyberbullying increases between ages 11 and 15 [56, 57], which is consistent with an increase in cell phone and internet-use in this age range [58]. The students in our study began in grade 9, who were mostly 15 years of age. From grade 9 through 12 we observed a fairly constant level of cyberbullying.

Older children in each school grade were least likely to be victims of bullying, which is again consistent with their having better social skills to better avoid being bullied [55]. On the other hand, younger children in each school grade were also less likely to be victims of bullying. It may be that younger students in a school grade are there because they are better students, have more supportive parents, and generally more connected with their teachers. Research has shown that adolescents with a higher level of school connectedness are at lower risk for bullying victimization [59, 60]. Adolescents who feel more connected with their school and have parents that are connected with the school are less likely to be victims of bullying [61–63].

We observed that the occurrence and frequency of being bullied and/or cyberbullied was positively associated with feelings of sadness and consideration or attempts of suicide. An association between bullying victimization and mental health problems has been observed previously in several studies [64–68]. Being cyberbullied was more strongly associated with the selected mental health problems than being bullied on school property, perhaps because victims of cyberbullying are less likely aware of their bully and feel more helpless against indirect bullying.

Some people are more likely to be bullied simply because they represent an easier option for bullying than if they belonged to the mainstream group [69]. In addition, such individuals may be less understood and viewed as different [70, 71]. For example, there are several examples where students are more likely bullied if their religious practices are less understood (e.g., Muslim girls wearing head scarves, Sikh boys wearing turbans, and Jewish boys wearing yarmulkes) [72]. Anti-Muslim and anti-Sikh bullying has increased in recent years as these groups have been linked with terrorism [72]. In a similar manner, students who use substances, who are sexually active, who have weight problems, or asthma, may be bullied simply because their behaviors or conditions are not widely accepted or understood, or because they appear more vulnerable.

Eating breakfast every day, being physically active, and playing on sports teams was associated with lower victimization from bullying. These behaviors are consistent with social norms that reflect better health, group protection, and lower vulnerability. On the other hand, playing video games an average of 3 or more hours per day was associated with increased risk of being bullied. These students may appear more vulnerable and prone to bullying because they are less likely to be involved in protective behaviors such as participating in sports or being connected to school. It may also be that they have less parental supervision. Data in the current study do not allow us to fully explain this result, but research has shown that lower parental education and broken families are associated with lower family income and less supervision, which, in turn, increase the risk of being bullied [73, 74]. Not living with a mother or father may also reflect a history of greater family conflict and less family attachment, which have further been shown to be associated with increased risk of being a victim of bullying [75].

Being a victim of bullying at school compared with cyberbullying was higher across the mental health (9–21 %), substance use (11–27 %), sexual behavior (8–28 %), weight (2–29 %), other behavior (28–31 %), and asthma (25 %) variables shown in Table 3. Yet the strength of the association (risk ratio) was generally greater between these variables and cyberbullying victimization. Therefore, although the variables tended to have a stronger association with being cyberbullied, the overall burden of the items was greater for bullying victimization at school because this form of bullying was more common.

The bullying questions used in this study were limited to those available in the YRBS. While the survey is deemed to be valid and reliable, and it is used throughout the United States, the available questions on bullying were limited, not allowing us to tease out specific types of bullying on school property or cyberbullying. In addition, the bullying questions asked about the past 12 months, whereas a shorter time period, such as that past 30 days, could have been more useful. Further, the YRBS survey did not include questions relating to teachers or other school staff members helping with bullying problems, such as feeling comfortable going to a teacher or other school staff member with a concern about bullying victimization.

## Conclusion

Females carry a greater risk of bullying victimization than males. Being bullied on school property decreases with later school grades, but cyberbullying remains constant for males and females within each racial/ethnic group. Maintaining closeness and intimacy in peer groups may be more important in females, causing them to be more likely to choose cyberbullying to remain anonymous. Blacks and Hispanics are less likely to be bullied, possibly because they appear less vulnerable or willing to be victimized. Lower levels of cyberbullying among Blacks and Hispanics corresponds with generally lower Internet and computer use. Occurrence and frequency of being bullied and/or cyberbullied is positively associated with feelings of sadness and consideration or attempts of suicide. Students who use substances, are sexually active, have weight problems, play video games an average of 3 or more hours per day, or have asthma are at greater risk of being bullied, perhaps because they represent an easier option for bullying as they do not belong to the mainstream group. Protective behaviors against bullying victimization include eating breakfast every day, being physically active, and playing on sports teams. These individuals may appear less vulnerable because of better health and group protection. Although being bullied on school property or cyberbullied are associated with greater risk of mental health problems, being both bullied on school property and cyberbullied carries the greatest risk.
